# Blood transfusion is correlated with elevated adult all-cause mortality and cardiovascular mortality in the United States: NHANES 1999 to 2018 population-based matched propensity score study

**DOI:** 10.1016/j.clinsp.2024.100379

**Published:** 2024-05-04

**Authors:** Jie Shi, Min Meng, Rina Sa, Lijun Yu, Yali Lu, Bei Gao

**Affiliations:** a Department of Blood Transfusion, The Second Hospital of Lanzhou University, Lanzhou, Gansu, China; b School Hospital of Lanzhou University, Lanzhou, Gansu, China; c Department of Medical, Gansu Provincial Hospital, Lanzhou, Gansu, China; d Department of Pharmacy, Gansu Provincial Hospital, Lanzhou, Gansu, China

**Keywords:** Blood transfusion, All-cause mortality, Cardiovascular mortality, Propensity Score-Matching

## Abstract

**Background and aims:**

The association of blood transfusion with an increase in medium- and short-term mortality in specific populations has been confirmed. However, the correlation between blood transfusion and long-term mortality in the general population remains unclear. This cohort study evaluated the correlation between blood transfusion and overall and cause-specific mortality in the general American adult population.

**Methods:**

The authors utilized 10 sets of 2-year cycle data (1999–2018) from the National Health and Nutrition Examination Survey on the outcomes of adults who did and did not receive blood transfusions. Propensity score-matching (1:1) was performed based on age, sex, race, education level, marital status, poverty-income ratio, arteriosclerotic cardiovascular disease, cancer, anemia, hypertension, and diabetes status. After controlling for demographic characteristics and clinical risk factors, Cox regression analysis was performed to evaluate the correlation between blood transfusion and all-cause and cause-specific mortality.

**Results:**

The study included 48,004 adult participants. The risk of all-cause mortality increased by 101 % with blood transfusion, and the risk of cardiovascular mortality increased by 165 %. After propensity score-matching, 6,116 pairs of cases were retained, and the risk of all-cause mortality increased by 84 % with blood transfusion, and the risk of cardiovascular mortality increased by 137 %. The sensitivity analysis results were robust.

**Conclusions:**

In the general American population, blood transfusion significantly impacts long-term all-cause and cardiovascular mortality and may be an unacknowledged risk factor for death. Thus, the effective management of blood transfusion in the general population may be beneficial.

## Highlights

• Blood transfusion significantly impacts long-term all-cause and cardiovascular mortality in the US population.
• After propensity score-matching, the risk of all-cause mortality increased by 78 % with blood transfusion, and the risk of cardiovascular mortality increased by 102 %.
• The effective management of blood transfusion in the general population may be beneficial.

## Introduction

Blood transfusion is mainly used to support patients undergoing surgery, those diagnosed with a critical illness, or those undergoing cancer treatment in high-income countries.^1^

Accumulating evidence indicates that blood transfusion is correlated with adverse outcomes such as increased morbidity and mortality.^2^ A study conducted in 2021 reported a correlation between a 3-fold increase in mortality rates within 60 days after surgery and intraoperative blood transfusion.^3^ Furthermore, blood transfusion has been linked to higher all-cause mortality in colorectal cancer,^4^ head and neck cancer,^5^ and COVID-19 patients.^6^

However, the relationship between blood transfusion and mortality remains unclear. Transfusion Requirements in Critical Care (TRICC) reported that, compared to a restrictive strategy of red-cell transfusion, patients with acute myocardial infarction and unstable angina who used a liberal strategy during hospitalization had a higher mortality rate; but for all critically ill patients, there no significant difference in the 30-day mortality rate between the two strategies.^7^ The Transfusion Requirements in Cardiac Surgery (TRICS) III trial also revealed that there was no evidence of a difference in the primary composite outcomes (all-cause death, myocardial infarction, stroke, etc.) between the restrictive or liberal strategy of red-cell transfusion for patients at moderate to high risk of death after cardiac surgery, either at discharge or at day 28 or 6 months follow-up.^8^^,^^9^ A retrospective cohort study performed in 1998 showed that perioperative blood transfusion did not affect 30-day and 90-day mortality rates after adjusting for multiple risk factors in consecutive hip fracture patients.^10^ A 2018 meta-analysis of patients undergoing major orthopedic surgery after perioperative blood transfusion administration reported no increase in 30-day post-surgery mortality rates in either the randomized controlled trials or observational studies.^11^ In these previous studies, the effect of blood transfusion on mortality usually focused on perioperative transfusion in certain types of surgery (cardiac or orthopedic surgeries, for instance) or in patients with malignant tumors. However, limited information is available on the all-cause mortality of blood transfusion in the general population. Additionally, since studies concentrated mainly on short-term (30–90 days) mortality,^3^^,^^10^^,^^11^ the long-term impact of blood transfusion remains unknown.

The authors therefore conducted a large cohort study based on the most recent data available from the National Health and Nutrition Examination Survey (NHANES). This study aimed to determine the association between blood transfusion and long-term mortality rates in the general US adult population.

## Methods

### Study population

The study was implemented across 10 sets of 2-year cycle datasets (beginning in 1999–2000 and ending in 2017–2018) of the NHANES. The NHANES is a widely used nationally representative dataset that links patient characteristics with mortality data. The methodological details of NHANES are available online (http://www.cdc.gov/nchs/nhanes.htm).

**Fig. 1:**
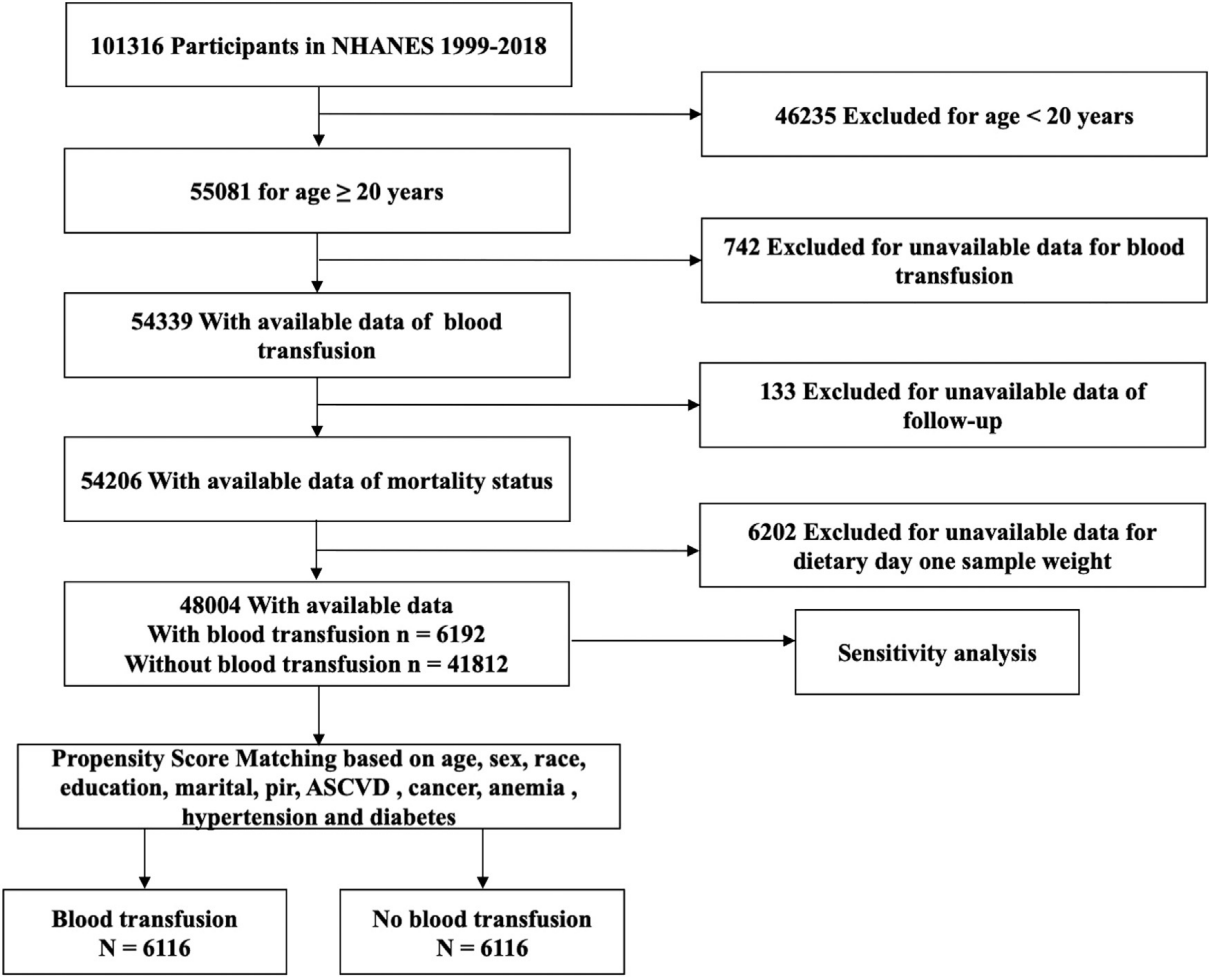
Flow diagram showing the selection of study participants. NHANES, National Health and Nutrition Examination Survey.

Of the 54,339 adults (aged ≥ 20 years) who underwent in-home interviews and had available data on blood transfusion, the authors excluded 133 individuals with missing follow-up data and 6202 individuals with unavailable data on dietary day one sample weight. The remaining 48,004 individuals were included in this study (Fig. 1). All participants provided informed consent. The National Centre for Health Statistics Research's Ethics Review Board approved the protocol for this study (protocol number: #98–12, #2005–06, #2011–17, and #2018–01). An observational study following STROBE guidelines was conducted in this study.

### Primary exposure: blood transfusion

Treatment with blood transfusion was determined based on self-reported data, in response to the question “Have you ever received a blood transfusion?”.

### Outcomes: All-Cause mortality and cause-specific mortality

Participants were matched with National Death Index records. Mortality data was obtained by tracking participants until December 31, 2019, which is the most recent available data. After examination, a follow-up period began until death or the conclusion of the follow-up. The 113 Underlying Cause of Death codes were used to recode all deaths into comparable ICD-10 classifications of deaths (see https://www.cdc.gov/nchs/data/datalinkage/public-use-linked-mortality-file-description.pdf). The main outcomes were overall and cause-specific mortality rates, including deaths from cardiovascular diseases and cancer. Cardiovascular mortality included death caused by heart disease or cerebrovascular diseases.

### Covariates

The study included covariates considered confounders according to existing literature and clinical judgment. Demographic information, including age, sex, race, education levels, marital status, and poverty-income ratio, was collected using questionnaires during home interviews. Age was dichotomized into ≥ 65 years and < 65 years. Race was classified into Mexican American, other Hispanic, non-Hispanic white, non-Hispanic black, and other races based on the NHANES classification method. Three levels of education were identified: high school or less, some college, and college graduates. Marital status included married, widowed/divorced/separated, and never married. The poverty-income ratio was used to measure income level and the values were categorized as ≤130 %, 131 % to < 185 %, and ≥ 185 %.^12^ Respondents were classified using their Body Mass Index (BMI) as either normal (BMI < 25 kg/m^2^), overweight (25 kg/m^2^ ≤ BMI < 30 kg/m^2^), and obese (BMI ≥ 30 kg/m^2^). A sedentary lifestyle was defined as a lack of activity, including walking, bicycling, household or yard tasks, muscle strengthening, work, or recreational activities, among others.^13^ Smoking status was classified as either never smoked, ex-smoker, or currently smoking. Participants were classified based on daily alcohol intake as non-drinkers, moderate drinkers (1–2 drinks for men and 1 drink for women), and heavy drinkers (> 2 drinks for men and > 1 drink for women).^14^ The transfusion year was divided into "before 1972″, "1972–1991″, and "1992 to present", because since 1971 and 1991, respectively, tests for hepatitis B surface antigen and hepatitis C viral antibodies were required to be included in donors blood screening as infection risk factors by the Food and Drug Administration to prevent the transmission of infectious pathogens through blood transfusion and ensure blood safety.^15^^,^^16^

Arteriosclerotic Cardiovascular Disease (ASCVD) includes coronary heart disease, angina, heart attack, or stroke.^17^ Cancer diagnoses were self-reported. Hypertension was defined as systolic blood pressure of ≥140 mmHg or diastolic blood pressure of ≥ 90 mmHg and based on self-reported diagnoses and treatments. Diabetes was diagnosed with fasting blood sugar level > 7.0 mmoL/L or glycated haemoglobin A1c values > 6.5 %, or through self-reported medical history and use of diabetes medications.^18^ Hyperlipidaemia was defined as total cholesterol ≥ 200 mg/dL, triglyceride ≥ 150 mg/dL, low-density lipoprotein cholesterol ≥ 130 mg/dL, or high-density lipoprotein cholesterol < 50 mg/dL (females) or < 4 mg/dL (males); or taking lipid-lowering medications.^19^ According to the WHO standards, anemia is divided into mild, moderate, and severe.^20^ The use of coagulation modifiers was assessed via the standardized questionnaire. Furthermore, the quartiles of Red blood cell Distribution Width (RDW) and Healthy Eating Index (HEI) 2010 were included as covariables. The HEI 2010 was derived from 24-hour recall-based measures of dietary components (range, 0–100; higher scores indicated healthier diets).

### Statistical analysis

Continuous variables were represented as the weighted mean ± standard error, while categorical variables were reported as percentages with 95 % Confidence Intervals (95 % CI). Baseline characteristics were compared using linear regression for continuous variables and the Chi-Square test for categorical variables. To balance confounders between the two groups, 1:1 potential Propensity Score-Matching (PSM) was performed with a caliper width of 0.02 and was matched using covariates including demographic (age, sex, race, education level, marital status, and poverty-income ratio) and comorbidity (ASCVD, cancer, anemia, hypertension, and diabetes). A Multivariate Cox proportional regression model was utilized to evaluate the relationship between blood transfusion and all-cause and cause-specific mortality rates. The confounding factors included in the adjusted model were age, sex, race, smoking status, alcohol intake, transfusion year, ASCVD, cancer, anemia, coagulation modifiers, and RDW quartile. In addition, given that some of the participants especially those who were also accompanied by some diseases, which themselves lead to increased mortality, the authors performed a sensitivity analysis using a multivariate Cox proportional regression model after eliminating individuals with severe diseases, such as ASCVD, cancer and anemia. Kaplan-Meier survival curves were conducted for all-cause and cardiovascular mortality rates based on blood transfusion status. A full set of data and graphs were used for statistical analysis using R Software 4.2.2 from www.r-project.org. Appropriate sampling weights were used to obtain valid estimates, considering a multistage probability sampling design for the NHANES. Statistical significance was set at 0.05.

## Results

### Characteristics of participants

The study cohort included 48,004 adult participants from 10 NHANES cycles. Among them, 6192 individuals received blood transfusion, while 41,812 did not (Fig. 1). The overall blood transfusion ratio was 11.3 (95 % CI 10.8–11.9) and the proportion of blood transfusion in those who died was more than three times higher than in those who survived (Supplemental Table 1). The average age of the study individuals was 47.0 years, 52 % were female, and 68.3 % were non-Hispanic white. The participants who had received blood transfusion were mostly older, female, non-Hispanic white, obese (BMI ≥ 30 kg/m^2^), led a non-sedentary lifestyle, and the year they received blood transfusion was 1992 to the present. They were less educated; had ASCVD, cancer, hypertension, diabetes, hyperlipidemia, or anemia; and used more coagulation modifiers. In fact, the prevalence of ASCVD, cancer, hypertension, diabetes, and anemia in participants who had received blood transfusion was almost twice or more than twice that of participants who had not received blood transfusion. Most of them were married or cohabitating, never smoked, and heavy drinkers; however, the proportions were slightly lower than those in the non-transfusion group. In comparison to those in the group that were not transfused, lymphocyte counts, hemoglobin levels, and energy intake per day were lower in the blood transfusion group, while RDW and HEI were higher (Table 1).

**Table 1:** General characteristics of the included participants based on a history of blood transfusion, (*n* = 48,004).

**Characters**	**Total (*n* = 48,004)**	**No (*n* = 41,812)**	**Yes (*n* = 6192)**	**p-value**
Age (years)	47.0 ± 0.2	45.4 ± 0.2	59.5 ± 0.3	<0.001
< 65	81.9 (79.1,84.7)	85.1 (84.5,85.8)	56.7 (54.9,58.6)	
≥ 65	18.1 (17.1,19.0)	14.9 (14.2,15.5)	43.3 (41.4,45.1)	
Sex, male (%)	48.0 (46.3,49.7)	49.3 (48.7, 49.9)	37.8 (36.2, 39.4)	<0.001
Race (%)				<0.001
Mexican American	8.2 (7.3, 9.2)	8.6 (7.5, 9.7)	5.4 (4.4, 6.5)	
Other Hispanic	5.4 (4.6, 6.2)	5.6(4.7, 6.4)	4.1 (3.1, 5.1)	
Non-Hispanic White	68.3 (64.2, 72.3)	67.6 (65.5, 69.7)	73.6 (71.3, 75.8)	
Non-Hispanic Black	11.3 (10.3, 12.3)	11.2 (10.1, 12.4)	11.7 (10.3, 13.1)	
Other races^a^	6.8 (6.2, 7.3)	7.0 (6.3, 7.6)	5.2 (4.3, 6.1)	
Education (%)				<0.001
High school or less	41.3 (39.4,43.2)	40.5 (39.2,41.9)	47.2 (45.1, 49.3)	
Some college	31.1 (29.8,32.5)	31.2 (30.4,31.9)	30.9 (29.1,32.7)	
College graduate	27.5 (25.8,29.2)	28.2 (26.8,29.6)	21.9 (20.1,23.8)	
Marital status (%)				<0.001
Married/Living with Partner	61.8 (59.1,64.6)	62.2 (61.1, 63.4)	58.7 (56.9, 60.6)	
Widowed/Divorced/Separated	18.6 (17.8, 19.5)	17.1 (16.5, 17.8)	30.4 (28.8, 32.1)	
Never married	18.4 (17.5, 19.3)	19.5 (18.5, 20.6)	9.6 (8.5, 10.7)	
Poverty-income ratio (%)				<0.001
< 130 %	20.4 (19.2, 21.5)	20.1 (19.0, 21.1)	22.6 (20.9,24.2)	
130 %–180 %	9.0 (8.5, 9.5)	8.8 (8.4, 9.3)	10.7 (9.7, 11.6)	
> 180 %	63.7 (60.9, 66.6)	64.4 (63.1, 65.7)	58.3 (56.2, 60.3)	
BMI (kg/m^2^)	28.8 ± 0.1	28.7 ± 0.1	29.4 ± 0.1	<0.001
< 25.0	30.8 (29.5, 32.1)	31.4 (30.5, 32.3)	26.0 (24.4, 27.5)	
25.0–29.9	32.9 (31.5, 34.3)	32.9 (32.1, 33.7)	32.9 (31.2, 34.6)	
≥ 30	35.1 (33.7, 36.6)	34.8 (33.9, 35.8)	37.6 (35.9, 39.4)	
Sedentary lifestyle (%)	77.9 (74.9, 80.8)	79.3 (78.5, 80.1)	66.3 (64.6, 68.0)	<0.001
Smoking status (%)				<0.001
Never smoked	53.5 (51.7, 55.4)	54.1 (53.1, 55.2)	48.9 (47.1, 50.8)	
Ex-smokers	24.8 (23.6, 26.0)	23.9 (23.2, 24.6)	31.7 (29.9, 33.4)	
Currently smoking	21.6 (20.5, 22.7)	21.9 (21.1, 22.7)	19.4 (17.9, 20.8)	
Alcohol intake (%)				<0.001
Non-drinkers	10.8 (9.9, 11.8)	10.5 (9.6, 11.5)	13.2 (12.0, 14.4)	
Moderate-drinkers	31.5 (30.0, 32.9)	31.6 (30.6, 32.6)	30.2 (28.3, 32.0)	
Heavy drinkers	41.3 (39.6, 42.9)	42.1 (41.2, 43.1)	34.5 (32.8, 36.1)	
Transfusion year				<0.001
Before 1972	2.6 (2.4, 2.9)	‒	23.3 (21.7,24.9)	
1972‒1991	3.7 (3.4, 4.0)	‒	33.0 (31.3, 34.8)	
1992 to present	4.7 (4.4, 5.0)	‒	41.1 (39.3, 42.9)	
ASCVD (%)	8.0 (7.5, 8.6)	6.2 (5.9, 6.6)	22.3 (20.7, 23.9)	<0.001
Cancer (%)	9.6 (9.1, 10.1)	8.0 (7.6, 8.4)	22.2 (20.8, 23.5)	<0.001
Anemia (%)				<0.001
No	90.5 (87.2, 93.8)	91.3 (90.8, 91.9)	84.0 (82.6, 85.3)	
Mild	4.4 (4.1, 4.7)	3.9 (3.6, 4.2)	8.3 (7.4, 9.2)	
Moderate	1.5 (1.4, 1.7)	1.3 (1.1, 1.4)	3.7 (3.0, 4.4)	
Severe	0.1 (0.1, 0.1)	0.1 (0.0, 0.1)	0.2 (0.0, 0.3)	
Hypertension (%)	37.2 (35.7, 38.7)	34.3 (33.4, 35.2)	60.0 (58.4, 61.6)	<0.001
Diabetes (%)	11.9 (11.3,12.4)	10.7 (10.2, 11.1)	21.2 (19.7, 22.7)	<0.001
Hyperlipidemia (%)	69.0 (66.3, 71.7)	68.0 (67.2, 68.9)	76.7 (75.2, 78.1)	<0.001
Coagulation modifiers (%)	3.9 (3.6, 4.2)	3.0 (2.8, 3.2)	11.1 (10.1,12.1)	<0.001
WBC (×10^9/L)	7.3 ± 0.0	7.3 ± 0.0	7.3 ± 0.0	0.400
NEUT (×10^9/L)	4.4 ± 0.0	4.4 ± 0.0	4.4 ± 0.0	0.100
LYM (×10^9/L)	2.2 ± 0.0	2.2 ± 0.0	2.1 ± 0.0	<0.001
PLT (×10^9/L)	254.0 ± 0.7	254.4 ± 0.7	251.5 ± 1.6	0.100
HGB (g/dL)	14.3 ± 0.0	14.3 ± 0.0	13.9 ± 0.0	<0.001
RDW (%)	13.0 ± 0.0	13.0 ± 0.0	13.5 ± 0.0	<0.001
Q1	31.9 (30.3, 33.5)	33.1 (32.0, 34.3)	22.1 (20.4, 23.7)	
Q2	30.0 (28.7, 31.4)	30.4 (29.7, 31.1)	27.0 (25.4, 28.6)	
Q3	18.2 (17.3, 19.2)	18.1 (17.4, 18.8)	19.3 (17.9, 20.6)	
Q4	19.9 (19.1, 20.7)	18.3 (17.6, 19.0)	31.7(29.9, 33.5)	
Energy intake (kcal/day)	2160.7 ± 7.0	2193.5 ± 7.3	1904.2 ± 15.0	<0.001
Healthy Eating Index	50.6 ± 0.2	50.5 ± 0.2	51.7 ± 0.3	<0.001
Q1	25.6 (24.3, 27.0)	26.1 (25.2, 27.0)	22.2 (20.6, 23.8)	
Q2	25.1 (24.0, 26.1)	25.1 (24.4, 25.8)	24.8 (23.1, 26.4)	
Q3	24.5 (23.5, 25.6)	24.4 (23.7, 25.0)	25.7 (24.3, 27.1)	
Q4	24.8 (23.6, 26.0)	24.5 (23.5, 25.5)	27.3 (25.6, 29.1)	

Values are weighted means ± standard error or weighted% (95 % Confidence Interval); p-values are weighted.

BMI, Body Mass Index; ASCVD, Arteriosclerotic Cardiovascular Disease; WBC, White Blood Cell Count; NEUT, Neutrophil Cell Count; LYM, Lymphocyte Cell Count; PLT, Platelet Count; HGB, Hemoglobin; RDW, Red Blood Cell Distribution Width.

a Other races contain Non-Hispanic Asian participants and other non-Hispanic race (including non-Hispanic multiracial).

### Characteristics of participants after psm

Following PSM, 6116 pairs of cases were analyzed in detail (Table 2). The overall blood transfusion ratio was increased to 49.4 (95 % CI 46.9–52.0). The proportion of blood transfusion in those who died was higher than that in those who survived and a statistical difference was found between individuals who died and those who survived (*p* < 0.001) (Supplemental Table 2). The results (Table 2) also showed that the two groups were comparable in age, sex, race, education level, marital status, poverty-income ratio, as well as BMI, smoking status, alcohol intake, ASCVD, cancer, anemia, hypertension, diabetes, hyperlipidemia, and HEI. After PSM, the average age of the individuals was 58.3 years, and females and non-Hispanic whites remained predominant. Compared to participants without blood transfusion, most participants who received blood transfusion had non-sedentary lifestyles, using coagulation modifiers, and the year they received blood transfusion was 1992 to the present. They had lower lymphocyte cell count, hemoglobin level, and energy intake per day but higher RDW.

**Table 2:** General characteristics of the included participants based on a history of blood transfusion after propensity score matching, (*n* = 12,232).

**Characters**	**Total (*n* = 12,232)**	**No (*n* = 6116)**	**Yes (*n* = 6116)**	**p-value**
Age (years)	58.3 ± 0.3	57.4 ± 0.4	59.3 ± 0.3	0.500
< 65	56.8 (54.0, 59.6)	56.3(54.3,58.3)	57.3 (55.4, 59.2)	
≥ 65	43.2 (40.6, 45.8)	43.7 (41.7, 45.7)	42.7 (40.8, 44.6)	
Sex, male (%)	38.2 (36.1, 40.3)	38.4 (36.8, 40.0)	38.0 (36.4, 39.6)	0.800
Race (%)				0.700
Mexican American	5.3 (4.5, 6.2)	5.2 (4.4, 6.0)	5.5 (4.4, 6.6)	
Other Hispanic	4.3 (3.4, 5.1)	4.5 (3.6, 5.4)	4.1 (3.1, 5.1)	
Non-Hispanic White	73.5 (68.5, 78.4)	73.5 (71.3, 75.7)	73.4 (71.2, 75.7)	
Non-Hispanic Black	11.9 (10.7, 13.0)	12.0 (10.5, 13.5)	11.7 (10.3, 13.2)	
Other races^a^	5.0 (4.4, 5.7)	4.8 (4.1, 5.6)	5.2 (4.3, 6.1)	
Education (%)				0.700
High school or less	47.3 (44.5, 50.1)	47.6 (45.5, 49.8)	46.9 (44.8, 49.0)	
Some college	30.5 (28.5, 32.5)	30.1 (28.1, 32.1)	31.0 (29.2, 32.8)	
College graduate	22.2 (20.5, 23.9)	22.3 (20.3, 24.3)	22.1 (20.2, 23.9)	
Marital status (%)				0.400
Married/Living with Partner	59.3 (55.9, 62.8)	59.8 (57.9, 61.6)	58.9 (57.0, 60.8)	
Widowed/Divorced/Separated	30.1 (28.3, 32.0)	30.1 (28.5, 31.8)	30.2 (28.5, 31.8)	
Never married	9.5 (8.6, 10.3)	9.3 (8.1, 10.4)	9.7 (8.6, 10.8)	
Poverty-income ratio (%)				0.900
< 130 %	22.3 (20.7, 23.9)	22.1 (20.5, 23.7)	22.6 (20.9, 24.3)	
130 %–180 %	10.6 (9.6, 11.5)	10.6 (9.6, 11.6)	10.5 (9.5, 11.5)	
> 180 %	58.9 (55.5, 62.2)	59.2 (57.2, 61.2)	58.5 (56.4, 60.7)	
BMI (kg/m^2^)	29.4 ± 0.1	29.5 ± 0.1	29.4 ± 0.1	0.700
< 25.0	26.1 (24.3, 27.8)	26.0 (24.4, 27.7)	26.1 (24.5, 27.6)	
25.0–29.9	33.3 (31.3, 35.4)	33.8 (32.2, 35.5)	32.8 (31.1, 34.5)	
≥ 30	38.1 (36.0, 40.2)	38.4 (36.6, 40.3)	37.7 (35.9, 39.4)	
Sedentary lifestyle (%)	68.7 (65.3, 72.1)	70.7 (69.0, 72.5)	66.6 (64.8, 68.3)	<0.001
Smoking status (%)				0.400
Never smoked	49.4 (46.9, 51.8)	49.9 (47.9, 51.9)	48.8 (46.9, 50.7)	
Ex-smokers	31.8 (29.9, 33.8)	32.0 (30.2, 33.9)	31.6 (29.9, 33.4)	
Currently smoking	18.8 (17.2, 20.3)	18.1 (16.5, 19.6)	19.5 (18.0, 21.0)	
Alcohol intake (%)				0.600
Non-drinkers	13.5 (12.3, 14.7)	13.9 (12.6, 15.3)	13.0 (11.8, 14.2)	
Moderate-drinkers	30.6 (28.6, 32.6)	30.9 (28.9,32.9)	30.3 (28.4, 32.1)	
Heavy drinkers	34.1 (32.0, 36.2)	33.6 (31.8, 35.4)	34.7 (33.0, 36.3)	
Transfusion year				<0.001
Before 1972	11.6 (10.6, 12.5)	‒	23.4 (21.8, 25.0)	
1972‒1991	16.4 (15.1, 17.7)	‒	33.2 (31.4, 35.0)	
1992 to present	20.2 (18.9, 21.5)	‒	40.9 (39.1,42.7)	
ASCVD (%)	21.7 (20.0, 23.3)	21.7 (20.3, 23.1)	21.6 (20.0,23.2)	0.900
Cancer (%)	21.6 (20.1, 23.1)	21.4 (19.9, 22.9)	21.7 (20.4, 23.1)	0.800
Anemia (%)				0.200
No	85.4 (81.1, 89.8)	86.4 (85.2, 87.6)	84.4 (83.1, 85.8)	
Mild	7.6 (7.0, 8.3)	7.1 (6.2, 8.0)	8.2 (7.3,9.1)	
Moderate	3.2 (2.8, 3.6)	3.0 (2.4, 3.5)	3.4 (2.8, 4.0)	
Severe	0.2 (0.1, 0.3)	0.1 (0.0, 0.3)	0.2 (0.0, 0.3)	
Hypertension (%)	60.2 (57.1, 63.4)	60.8 (59.1, 62.6)	59.6 (58.0, 61.3)	0.300
Diabetes (%)	20.7 (19.4, 22.0)	20.8 (19.5, 22.0)	20.6 (19.1, 22.1)	0.900
Hyperlipidemia (%)	77.1 (73.3, 80.9)	77.6 (76.2, 79.0)	76.6 (75.1, 78.0)	0.300
Coagulation modifiers (%)	10.0 (9.1, 10.9)	9.2 (8.2, 10.1)	10.8 (9.8, 11.8)	<0.001
WBC (×10^9/L)	7.3 ± 0.0	7.4 ± 0.1	7.3 ± 0.0	0.200
NEUT (×10^9/L)	4.4 ± 0.0	4.4 ± 0.0	4.4 ± 0.0	0.600
LYM (×10^9/L)	2.1 ± 0.0	2.2 ± 0.0	2.1 ± 0.0	0.010
PLT (×10^9/L)	251.9 ± 1.1	252.1 ± 1.3	251.7 ± 1.6	0.800
HGB (g/dL)	14.0 ± 0.0	14.0 ± 0.0	13.9 ± 0.0	< 0.001
RDW (%)	13.3 ± 0.0	13.2 ± 0.0	13.5 ± 0.0	<0.001
Q1	24.6 (22.9, 26.2)	26.8 (25.1, 28.5)	22.2 (20.6, 23.9)	
Q2	28.0 (26.2, 29.9)	29.0 (27.3, 30.7)	27.0 (25.4, 28.7)	
Q3	19.2 (17.8, 20.6)	19.1 (17.6, 20.6)	19.3 (17.9, 20.7)	
Q4	28.2 (26.8, 29.6)	25.0 (23.8, 26.3)	31.4 (29.7, 33.2)	
Energy intake (kcal/day)	1942.8 ± 12.9	1975.0 ± 18.1	1910.0 ± 15.0	0.002
Healthy Eating Index	51.9 ± 0.2	52.0 ± 0.3	51.7 ± 0.3	0.400
Q1	22.8 (21.1, 24.4)	23.3 (21.7, 24.9)	22.3 (20.7, 23.9)	
Q2	24.0 (22.4, 25.6)	23.2 (21.6, 24.8)	24.8 (23.1, 26.4)	
Q3	25.3 (23.7, 27.0)	25.0 (23.2, 26.8)	25.7 (24.3, 27.1)	
Q4	27.9 (26.2, 29.7)	28.5 (26.7, 30.3)	27.3 (25.5, 29.1)	

Values are weighted means ± standard error or weighted% (95 % Confidence Interval); p-values are weighted.

BMI, Body Mass Index; ASCVD, Arteriosclerotic Cardiovascular Disease; WBC, White Blood cell Count; NEUT, Neutrophil Cell Count; LYM, Lymphocyte Cell Count; PLT, Platelet Count; HGB, Hemoglobin; RDW, Red blood cell Distribution Width.

a Other races contain Non-Hispanic Asian participants and other non-Hispanic race (including non-Hispanic multiracial).

### Association between blood transfusion with all-cause mortality and cause-specific mortality

Overall, 2082 participants with blood transfusion died, including 675 cardiovascular deaths and 421 cancer deaths after a 9.3-year median follow-up (maximum follow-up, 20.8 years). After adjusting for demographic variables and potential risk factors, blood transfusion was associated with an increased all-cause mortality rate (HR = 2.06; 95 % CI 1.52–2.78) (*p* < 0.001) and increased cardiovascular mortality rate (HR = 2.86; 95 % CI 1.77–4.62) (*p* < 0.001). Conversely, the correlation between blood transfusion and cancer-related mortality was attenuated (*p* > 0.05) (Table 3).

**Table 3:** Associations between blood transfusion and all-cause and cause-specific mortality among adults in NHANES 1999–2018.

	**Deaths**	**Unadjusted model**		**Model 1**		**Model 2**	
	**n/N**	**HR (95 % CI)**	**p**	**HR (95 % CI)**	**p**	**HR (95 % CI)**	**p**
**Before PSM**							
All-cause mortality	2135/7448	3.58 (3.33, 3.86)	<0.001	1.87 (1.73, 2.03)	<0.001	2.06 (1.52, 2.78)	<0.001
Cardiovascular mortality	693/2319	4.64 (4.10, 5.25)	<0.001	2.13 (1.86, 2.44)	<0.001	2.86(1.77,4.62)	<0.001
Cancer mortality	427/1680	3.17 (2.74, 3.67)	<0.001	1.79 (1.55, 2.07)	<0.001	0.88 (0.41, 1.87)	0.740
**After PSM**							
All-cause mortality	2082/3864	1.31 (1.21, 1.43)	<0.001	1.36 (1.24, 1.49)	<0.001	1.84 (1.33, 2.55)	<0.001
Cardiovascular mortality	675/1282	1.38 (1.20, 1.60)	<0.001	1.44 (1.25, 1.66)	<0.001	2.37 (1.41, 3.98)	0.001
Cancer mortality	421/779	1.36 (1.14, 1.63)	<0.001	1.42 (1.19, 1.71)	<0.001	0.90 (0.40, 2.04)	0.800
**Sensitivity analysis after exclusion of participants with severe diseases (None, *n* = 35,130)**							
All-cause mortality	701/3490	3.01 (2.65, 3.41)	<0.001	1.74 (1.51,2.01)	<0.001	2.14 (1.24, 3.70)	0.010
Cardiovascular mortality	207/1004	3.55 (2.86, 4.40)	<0.001	1.84 (1.43, 2.37)	<0.001	3.85 (1.65, 8.99)	0.002
Cancer mortality	138/793	2.43 (1.85, 3.19)	<0.001	1.61 (1.20, 2.15)	0.001	1.37 (0.34, 5.43)	0.660

Model 1, Adjusted for: sex, age, race; Model 2, Adjusted for: sex, age, race, smoking status, alcohol intake, transfusion year; ASCVD; Cancer, Anemia, Coagulation modifiers and red blood cell distribution width quartile.

NHANES, National Health and Nutrition Examination Survey; HR, Hazard Ratio; 95 % CI, 95 % Confidence Interval; ASCVD, Arteriosclerotic Cardiovascular Disease.

After PSM, during the 10.8-year median follow-up, 2082 participants with blood transfusion died, including 675 cardiovascular deaths, and 421 cancer deaths. After PSM (Table 3), Hazard Ratios (HR) for all-cause mortality, cardiovascular mortality, and cancer mortality in participants who received blood transfusion were 1.31, 1.38, and 1.36 respectively in unadjusted models. In the final adjusted model, blood transfusion was associated with an increased all-cause mortality rate (HR = 1.84; 95 % CI 1.33–2.55) (*p* < 0.001) and increased cardiovascular mortality rate (HR = 2.37; 95 % CI 1.41–3.98) (*p* = 0.010). Conversely, the association between blood transfusion and cancer-related mortality was attenuated (*p* > 0.05). Additionally, the sensitivity analysis of the present results was robust, and these results were consistent with the data presented above, further supporting these conclusions.

**Fig. 2:**
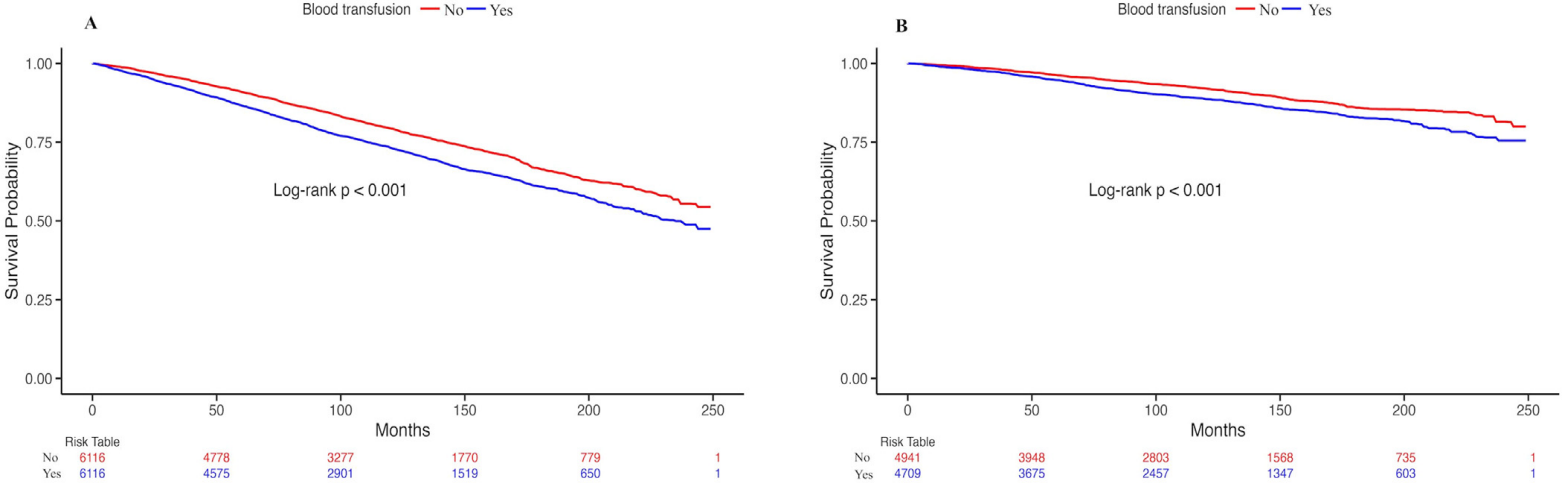
Kaplan-Meier survival curve of the blood transfusion group and the non-transfusion group. (A) All-cause mortality; (B) Cardiovascular mortality.

The Kaplan-Meier curve (Fig. 2) revealed a significant increase in all-cause and cardiovascular mortality rates in the blood transfusion group compared to those in the non-transfused group (*p* < 0.001).

## Discussion

In the general population, blood transfusion positively correlated with all-cause mortality and cardiovascular mortality, even after adjusting for confounding factors and conducting the sensitivity analysis. Herein, the authors utilized nationally representative data over a 20-year period, allowing generalization to adults non-institutionalized US adults with a sufficient follow-up period (median 10.8-years). Notably, the authors implemented the PSM^21^ for bias reduction and ensured a sufficient sample size (6116 patients per group, representing 48.6 million noninstitutionalized residents of the United States). Meanwhile, the authors considered adjustments for coagulation modifiers and multiple comorbidities. Additionally, the authors also carried out sensitivity analysis to avoid the influence of some serious diseases on the mortality rate, in order to draw more objective conclusions.

At the 6-month follow-up of patients after cardiac surgery, although there were no statistically significant results between a restrictive or liberal strategy of red-cell transfusion in the TRICS III trial, the main composite outcomes of the two strategies were as high as 17.4 % and 17.1 %, respectively, which still deserve attention, suggesting that the authors should further explore the correlation between blood transfusion itself and all-cause mortality rate, myocardial infarction, stroke, etc.^9^ A meta-analysis of perioperative blood transfusion in cardiac surgery patients (median follow-up, 6.4 years) showed increased mortality risks (log of the HR = 0.45; 95 % CI 0.30–0.60; *p* < 0.001).^22^ A cohort study confirmed an increased 1-year mortality rate (HR = 1.21; 95 % CI 1.06–1.46) among intensive care unit survivors who received transfusions compared to non-transfusion group,^23^ suggesting that blood transfusion may be strongly correlated with increased long-term mortality, which is consistent with the present findings. Nonetheless, most previous studies have been conducted in clinical populations, paying little attention to the causes of death. Due to the lack of long-term follow-up data, studies on the correlation between blood transfusion and all-cause and cause-specific mortality in the general population are lacking. The present findings suggest that blood transfusion may be a standalone risk factor for increased death rate among US adults.

This study found a positive correlation between blood transfusion and cardiovascular mortality in the general population. A meta-analysis of 203,665 participants from 1966 to 2012, showed that blood transfusion increased all-cause mortality (18.2% vs. 10.2 % in non-blood transfusion treatment) for myocardial infarction patients.^24^ Moreover, blood transfusion was reported to increase the risk of repeated myocardial infarctions and long-term/short-term mortality, especially among individuals with ST-segment elevation myocardial infarction.^25^^,^^26^ Moreover, a study of 19,680 patients undergoing major abdominal surgery showed an increased myocardial infarction incidence 72 h after the administration of perioperative blood transfusion (*p* = 0.002).^27^ These results, along with the present research findings, could be explained as follows. First, red blood cells are crucial to both physiological hemostasis and pathological thrombosis as evidenced by mechanistic studies.^28^^,^^29^ Second, the inflammatory and immunomodulatory responses to red blood cell transfusion may further exacerbate hypercoagulability.^30^ Third, red blood cell infusion leads to chronic low-grade inflammation medicated by immune factors, which is closely related to the metabolic triad of obesity, diabetes, and cardiovascular disease.^22^ Fourth, more women than men received a blood transfusion in this study, and they had higher rates of ASCVD, hypertension, diabetes, hyperlipidemia, and anemia, which may lead to poor long-term outcomes.

However, due to the use of cross-sectional survey data, this study had several limitations. First, self-reported blood transfusion exposure led to the lack of indications for transfusion and detailed transfusion information, which hindered further analysis such as comparative studies. The authors could not differentiate restrictive red blood cell transfusion strategy from liberal red blood cell transfusion strategy, nor could the authors determine whether red blood cells, platelets, or plasma are being infused, as well as the infusion unit or dosage, but this information may be better used to analyze outcomes and develop appropriate interventions. This may also be the reason why the present research results were inconsistent with the results of the MINT trial,^31^ which suggested that there was no significant difference in the 30 days mortality rate between the restrictive-strategy group and the liberal-strategy group for patients with myocardial infarction and hemoglobin levels < 10 g/dL. Second, incomplete data hindered the conduction of some of the planned subgroups. Third, inability to establish causality between blood transfusion and mortality due to the study's observational nature. Fourth, the authors were unable to distinguish patients who had cardiac surgery from those who had non-cardiac surgery in this observational study. A large number of cardiac surgery patients may be excluded from the sensitivity analysis, which may lead to selection bias. Fifth, in spite of the fact that the authors adjusted the analyses for important confounding factors, including serious illnesses related to death, residual confounding may still exist if factors are unknown or unmeasured. These defects can be overcome in the future through more well-designed randomized controlled trials.

## Conclusion

Blood transfusion significantly impacts long-term all-cause mortality and cardiovascular mortality rates in the general US population, potentially being an under-recognized risk factor for death. Thus, the effective management of blood transfusion in the general population may be beneficial. However, further studies, including subgroup analysis, are warranted to define the correlation between blood transfusion and mortality in specific populations.

## Data availability statement

Publicly available datasets were analyzed in this study. This data can be found here: https://wwwn.cdc.gov/nchs/nhanes/.

## Ethics statement

The studies involving human participants were reviewed and approved by the National Center for Health Statistics. The patients/participants provided written informed consent to participate in this study.

## Authors’ contributions

J.S. and B.G. contributed to the conception and design, the acquisition, analysis, and interpretation of the data, the drafting of the article, or critical revision of important intellectual content. J.S., R-N.S. and B.G. collected and analyzed data. M. M., l-J.Y. and Y-L.L. reviewed the article and offered the critical revision for important intellectual content. J.S. received financial support. All authors approved the final version and agreed to be accountable for all aspects of the work.

## Declaration of competing interest

The authors declare the following financial interests/personal relationships which may be considered as potential competing interests: J.S. reports financial support was provided by the Science and Technology Department of Gansu Province.

## Appendix Supplementary materials

Image, application 1
