# Supplementary material for: Blood transfusion is correlated with elevated adult all-cause mortality and cardiovascular mortality in the United States: NHANES 1999 to 2018 population-based matched propensity score study
Source: Clinics (Sao Paulo). 2024 May 4;79:100379. doi: 10.1016/j.clinsp.2024.100379 (PMC11087911; doi:10.1016/j.clinsp.2024.100379)
Supplement: Supplementary file 1 [file mmc1.docx]

**CLINICS-D-23-00445_Supplementary Material**

**Supplemental Table 1** General characteristics of the included participants based on survival state, (n = 48004).

| **Characters** | **Total**  **(n = 48004)** | **Alive**  **(n = 40556)** | **Deceased**  **(n = 7448)** | **p-value** |
| --- | --- | --- | --- | --- |
| Age (years) | 47.0 ± 0.2 | 44.5 ± 0.2 | 66.0 ± 0.3 | <0.001 |
| < 65 | 81.9 (79.1, 84.7) | 87.4 (86.8, 88.0) | 39.4 (37.6, 41.2) |  |
| ≥ 65 | 18.1 (17.1, 19.0) | 12.6 (12.0, 13.2) | 60.6 (58.8, 62.4) |  |
| Sex, male (%) | 48.0 (46.3, 49.7) | 47.5 (46.9, 48.0) | 52.0 (50.7, 53.2) | <0.001 |
| Race (%) |  |  |  | <0.001 |
| Mexican American | 8.2 (7.3, 9.2) | 8.8 (7.7, 9.9) | 4.0 (3.1, 4.9) |  |
| Other Hispanic | 5.4 (4.6, 6.2) | 5.7 (4.9, 6.5) | 3.3 (1.9, 4.7) |  |
| Non-Hispanic White | 68.3 (64.2, 72.3) | 67.1 (65.0, 69.2) | 77.5 (75.2, 79.7) |  |
| Non-Hispanic Black | 11.3 (10.3, 12.3) | 11.2 (10.1, 12.4) | 11.7 (10.2, 13.1) |  |
| Other races^a^ | 6.8 (6.2, 7.3) | 7.2 (6.5, 7.8) | 3.6 (2.9, 4.3) |  |
| Education (%) |  |  |  | <0.001 |
| High school or less | 41.3 (39.4, 43.2) | 39.1 (37.7, 40.4) | 58.5 (56.6, 60.4) |  |
| Some college | 31.1 (29.8, 32.5) | 31.8 (31.0, 32.6) | 26.0 (24.5, 27.5) |  |
| College graduate | 27.5 (25.8, 29.2) | 29.1 (27.6, 30.5) | 15.3 (13.9, 16.7) |  |
| Marital status (%) |  |  |  | <0.001 |
| Married/Living with Partner | 61.8 (59.1, 64.6) | 63.0 (61.9, 64.2) | 52.7 (50.6, 54.8) |  |
| Widowed/Divorced/Separated | 18.6 (17.8, 19.5) | 16.3 (15.7, 16.9) | 36.5 (34.8, 38.1) |  |
| Never married | 18.4 (17.5, 19.3) | 19.7 (18.7, 20.7) | 8.4 (7.3, 9.5) |  |
| Poverty-income ratio (%) |  |  |  | <0.001 |
| < 130% | 20.4 (19.2, 21.5) | 19.6 (18.6, 20.7) | 26.0 (24.0, 27.9) |  |
| 130%–180% | 9.0 (8.5, 9.5) | 8.5 (8.0, 8.9) | 13.3 (12.1, 14.5) |  |
| > 180% | 63.7 (60.9, 66.6) | 65.2 (63.9, 66.5) | 52.5 (50.5, 54.5) |  |
| BMI (kg/m^2^) | 28.8 ± 0.1 | 28.8 ± 0.1 | 28.7 ± 0.1 | <0.001 |
| < 25.0 | 30.8 (29.5, 32.1) | 30.9 (30.0, 31.9) | 29.5 (28.2, 30.8) |  |
| 25.0–29.9 | 32.9 (31.5, 34.3) | 32.9 (32.2, 33.7) | 32.5 (31.0, 34.0) |  |
| ≥ 30 | 35.1 (33.7, 36.6) | 35.4 (34.4, 36.3) | 33.4 (31.9, 35.0) |  |
| Sedentary lifestyle (%) | 77.9(74.9,80.8) | 80.4 (79.7, 81.2) | 58.1 (56.3, 60.0) | <0.001 |
| Smoking status (%) |  |  |  | <0.001 |
| Never smoked | 53.5 (51.7, 55.4) | 55.5 (54.4, 56.5) | 38.8 (37.2, 40.4) |  |
| Ex-smokers | 24.8 (23.6, 26.0) | 23.2 (22.4, 23.9) | 37.3 (35.7, 38.9) |  |
| Currently smoking | 21.6 (20.5, 22.7) | 21.3 (20.5, 22.1) | 23.8 (22.4, 25.3) |  |
| Alcohol intake (%) |  |  |  | <0.001 |
| Non-drinkers | 10.8 (9.9, 11.8) | 10.3 (9.4, 11.2) | 15.0 (13.6, 16.3) |  |
| Moderate-drinkers | 31.5 (30.0, 32.9) | 32.0 (31.0, 33.0) | 27.2 (25.5, 28.9) |  |
| Heavy drinkers | 41.3 (39.6, 42.9) | 42.6 (41.7, 43.5) | 31.0 (29.3, 32.7) |  |
| History of transfusion (%) | 11.3 (10.8, 11.9) | 9.1 (8.7, 9.5) | 28.5 (27.1,30.0) | <0.001 |
| Transfusion year |  |  |  | <0.001 |
| Before 1972 | 2.6 (2.4, 2.9) | 2.0 (1.8, 2.1) | 7.9 (7.1, 8.8) |  |
| 1972‒1991 | 3.7 (3.4, 4.0) | 3.3 (3.0,3.5) | 7.5 (6.8, 8.3) |  |
| 1992 to present | 4.7 (4.4, 5.0) | 3.7 (3.4, 4.0) | 12.0 (11.1, 12.8) |  |
| ASCVD (%) | 8.0 (7.5, 8.6) | 5.5 (5.2, 5.9) | 27.4 (25.7, 29.0) | <0.001 |
| Cancer (%) | 9.6 (9.1, 10.1) | 8.0 (7.6, 8.4) | 22.2 (21.0, 23.5) | <0.001 |
| Anemia (%) |  |  |  | <0.001 |
| No | 90.5 (87.2, 93.8) | 91.5 (91.0, 92.0) | 82.5 (81.1, 83.9) |  |
| Mild | 4.4 (4.1, 4.7) | 3.7 (3.4, 4.0) | 9.6(8.5,10.6) |  |
| Moderate | 1.5 (1.4, 1.7) | 1.3 (1.2, 1.5) | 3.1 (2.6, 3.6) |  |
| Severe | 0.1 (0.1, 0.1) | 0.1 (0.0, 0.1) | 0.1 (0.0, 0.2) |  |
| Hypertension (%) | 37.2 (35.7, 38.7) | 33.3 (32.5, 34.1) | 67.5 (65.9, 69.2) | <0.001 |
| Diabetes (%) | 11.9 (11.3, 12.4) | 10.0 (9.5, 10.4) | 26.5 (25.1, 27.8) | <0.001 |
| Hyperlipidemia (%) | 69.0 (66.3, 71.7) | 67.9 (67.0, 68.7) | 77.9 (76.6, 79.3) | <0.001 |
| Coagulation modifiers (%) | 3.9 (3.6, 4.2) | 2.6 (2.4, 2.8) | 14.2 (13.1, 15.2) | <0.001 |
| WBC (×10^9/L) | 7.3 ± 0.0 | 7.3 ± 0.0 | 7.5 ± 0.0 | <0.001 |
| NEUT (×10^9/L) | 4.4 ± 0.0 | 4.3 ± 0.0 | 4.6 ± 0.0 | <0.001 |
| LYM (×10^9/L) | 2.2 ± 0.0 | 2.2 ± 0.0 | 2.1 ± 0.0 | 0.003 |
| PLT (×10^9/L) | 254.0 ± 0.7 | 254.7 ± 0.7 | 249.3 ± 1.3 | <0.001 |
| HGB (g/dL) | 14.3 ± 0.0 | 14.3 ± 0.0 | 14.1 ± 0.0 | <0.001 |
| RDW (%) | 13.0 ± 0.0 | 13.0 ± 0.0 | 13.3 ± 0.0 | <0.001 |
| Q1 | 31.9 (30.3, 33.5) | 32.9 (31.7, 34.1) | 23.9 (22.7, 25.1) |  |
| Q2 | 30.0 (28.7, 31.4) | 30.0 (29.2, 30.7) | 30.3 (29.1, 31.6) |  |
| Q3 | 18.2 (17.3, 19.2) | 18.3(17.6, 19.0) | 17.6 (16.6, 18.7) |  |
| Q4 | 19.9 (19.1, 20.7) | 18.8 (18.1, 19.5) | 28.1 (26.7, 29.6) |  |
| Energy intake (kcal/day) | 2160.7 ± 7.0 | 2195.7 ± 7.3 | 1891.0 ± 17.5 | <0.001 |
| Healthy Eating Index | 50.6 ± 0.2 | 50.5 ± 0.2 | 51.7 ± 0.3 | <0.001 |
| Q1 | 25.6 (24.3, 27.0) | 26.1 (25.2, 27.0) | 22.0 (20.6, 23.3) |  |
| Q2 | 25.1 (24.0, 26.1) | 25.1 (24.4, 25.8) | 24.7 (23.1, 26.2) |  |
| Q3 | 24.5 (23.5, 25.6) | 24.2(23.5, 24.8) | 27.1 (25.6, 28.5) |  |
| Q4 | 24.8 (23.6, 26.0) | 24.6 (23.6, 25.6) | 26.3 (24.7, 27.9) |  |

Values are weighted means ± standard error or weighted % (95% Confidence Interval); p-values are weighted.

^a^ Other races contain Non-Hispanic Asian participants and other non-Hispanic race (including non-Hispanic multiracial).

BMI, Body Mass Index; ASCVD, Arteriosclerotic Cardiovascular Disease; WBC, White Blood Cell Count; NEUT, Neutrophil Cell Count; LYM, Lymphocyte Cell Count; PLT, Platelet Count; HGB, Hemoglobin; RDW, Red blood cell Distribution width.

**Supplemental Table 2** General characteristics of the included participants based on survival state after propensity score matching, (n = 12232).

| **Characters** | **Total**  **(n = 12232)** | **Alive**  **(n = 8368)** | **Deceased**  **(n = 3864)** | **p-value** |
| --- | --- | --- | --- | --- |
| Age (years) | 58.3 ± 0.3 | 54.1 ± 0.3 | 70.5 ± 0.3 | <0.001 |
| < 65 | 56.8 (54.0, 59.6) | 68.0 (66.5, 69.5) | 24.6 (22.3, 26.9) |  |
| ≥ 65 | 43.2 (40.6, 45.8) | 32.0 (30.5, 33.5) | 75.4 (73.1, 77.7) |  |
| Sex, male (%) | 38.2 (36.1, 40.3) | 35.9 (34.5, 37.3) | 44.7 (42.9, 46.6) | <0.001 |
| Race (%) |  |  |  | <0.001 |
| Mexican American | 5.3 (4.5, 6.2) | 6.1 (5.1, 7.0) | 3.3 (2.3, 4.2) |  |
| Other Hispanic | 4.3 (3.4, 5.1) | 4.9 (4.0, 5.8) | 2.5 (1.3, 3.8) |  |
| Non-Hispanic White | 73.5 (68.5, 78.4) | 70.7 (68.5, 72.9) | 81.4 (79.1, 83.7) |  |
| Non-Hispanic Black | 11.9(10.7,13.0) | 12.5 (11.1, 14.0) | 10.0 (8.5, 11.5) |  |
| Other races^a^ | 5.0 (4.4, 5.7) | 5.8 (5.0, 6.6) | 2.8 (2.0, 3.6) |  |
| Education (%) |  |  |  | <0.001 |
| High school or less | 47.3 (44.5, 50.1) | 42.4 (40.6, 44.3) | 61.1 (58.7, 63.6) |  |
| Some college | 30.5 (28.5, 32.5) | 32.3 (30.7, 33.9) | 25.4 (23.4, 27.5) |  |
| College graduate | 22.2 (20.5, 23.9) | 25.2 (23.3, 27.1) | 13.4 (11.8, 15.0) |  |
| Marital status (%) |  |  |  | <0.001 |
| Married/Living with Partner | 59.3 (55.9, 62.8) | 62.6 (61.0, 64.2) | 50.0 (47.3, 52.7) |  |
| Widowed/Divorced/Separated | 30.1 (28.3, 32.0) | 25.8 (24.5, 27.1) | 42.6 (40.2, 45.0) |  |
| Never married | 9.5 (8.6, 10.3) | 10.9 (9.8, 12.0) | 5.3 (4.1, 6.4) |  |
| Poverty-income ratio (%) |  |  |  | <0.001 |
| < 130% | 22.3 (20.7, 23.9) | 20.8 (19.4, 22.1) | 26.8 (24.6, 29.1) |  |
| 130%–180% | 10.6 (9.6, 11.5) | 9.0 (8.1, 9.8) | 15.1 (13.5, 16.7) |  |
| > 180% | 58.9 (55.5, 62.2) | 62.0 (60.1, 63.9) | 49.9 (47.2, 52.5) |  |
| BMI (kg/m^2^) | 29.4 ± 0.1 | 29.7 ± 0.1 | 28.7 ± 0.1 | <0.001 |
| < 25.0 | 26.1 (24.3, 27.8) | 25.1 (23.7, 26.6) | 28.7 (27.1, 30.3) |  |
| 25.0–29.9 | 33.3 (31.3, 35.4) | 33.5 (32.0, 35.0) | 32.7 (30.6, 34.8) |  |
| ≥ 30 | 38.1 (36.0, 40.2) | 39.9 (38.3, 41.5) | 32.7 (30.6, 34.7) |  |
| Sedentary lifestyle (%) | 68.7 (65.3, 72.1) | 74.4 (73.2, 75.6) | 52.4 (50.0, 54.8) | <0.001 |
| Smoking status (%) |  |  |  | <0.001 |
| Never smoked | 49.4 (46.9, 51.8) | 52.4 (50.8, 54.0) | 40.7 (38.1, 43.2) |  |
| Ex-smokers | 31.8 (29.9, 33.8) | 28.5 (27.0, 30.0) | 41.5 (39.1, 43.8) |  |
| Currently smoking | 18.8 (17.2, 20.3) | 19.1 (17.7, 20.4) | 17.9 (15.8, 19.9) |  |
| Alcohol intake (%) |  |  |  | <0.001 |
| Non-drinkers | 13.5 (12.3, 14.7) | 12.1 (10.9, 13.2) | 17.6 (15.6, 19.6) |  |
| Moderate-drinkers | 30.6 (28.6, 32.6) | 31.9 (30.2, 33.6) | 26.8 (24.7, 29.0) |  |
| Heavy drinkers | 34.1 (32.0, 36.2) | 36.8 (35.4, 38.3) | 26.3 (24.2, 28.4) |  |
| History of transfusion (%) | 49.4 (46.9, 52.0) | 47.6 (46.2, 49.0) | 54.6 (52.4, 56.7) | <0.001 |
| Transfusion year |  |  |  | <0.001 |
| Before 1972 | 11.6 (10.6, 12.5) | 10.3 (9.4, 11.1) | 15.3 (13.7, 16.8) |  |
| 1972‒1991 | 16.4 (15.1, 17.7) | 17.1 (15.8, 18.3) | 14.5 (13.1, 15.9) |  |
| 1992 to present | 20.2 (18.9, 21.5) | 19.3 (18.2, 20.5) | 22.6 (21.1, 24.2) |  |
| ASCVD (%) | 21.7 (20.0, 23.3) | 15.6 (14.6, 16.6) | 39.1 (36.6, 41.5) | <0.001 |
| Cancer (%) | 21.6 (20.1, 23.1) | 18.9 (17.6, 20.1) | 29.3 (27.7, 31.0) | <0.001 |
| Anemia (%) |  |  |  | <0.001 |
| No | 85.4 (81.1, 89.8) | 87.5 (86.5, 88.5) | 79.5 (77.6, 81.3) |  |
| Mild | 7.6 (7.0, 8.3) | 6.0 (5.3, 6.8) | 12.3 (10.9, 13.7) |  |
| Moderate | 3.2 (2.8, 3.6) | 3.0 (2.5, 3.4) | 3.7 (3.0, 4.5) |  |
| Severe | 0.2 (0.1, 0.3) | 0.2 (0.1, 0.3) | 0.0 (0.0, 0.1) |  |
| Hypertension (%) | 60.2 (57.1, 63.4) | 54.4 (52.9, 55.9) | 77.0 (75.0, 79.0) | <0.001 |
| Diabetes (%) | 20.7 (19.4, 22.0) | 17.5 (16.3, 18.6) | 29.9 (28.0, 31.8) | <0.001 |
| Hyperlipidemia (%) | 77.1 (73.3, 80.9) | 76.1 (74.8, 77.4) | 80.0 (78.3, 81.6) | <0.001 |
| Coagulation modifiers (%) | 10.0 (9.1, 10.9) | 7.1 (6.4, 7.9) | 18.2 (16.6, 19.7) | <0.001 |
| WBC (×10^9/L) | 7.3 ± 0.0 | 7.3 ± 0.0 | 7.5 ± 0.1 | 0.050 |
| NEUT (×10^9/L) | 4.4 ± 0.0 | 4.3 ± 0.0 | 4.6 ± 0.0 | <0.001 |
| LYM (×10^9/L) | 2.1 ± 0.0 | 2.2 ± 0.0 | 2.0 ± 0.0 | 0.020 |
| PLT (×10^9/L) | 251.9 ± 1.1 | 254.1 ± 1.3 | 245.6 ± 1.7 | <0.001 |
| HGB (g/dL) | 14.0 ± 0.0 | 14.0 ± 0.0 | 13.8 ± 0.0 | <0.001 |
| RDW (%) | 13.3 ± 0.0 | 13.3 ± 0.0 | 13.5 ± 0.0 | <0.001 |
| Q1 | 24.6 (22.9, 26.2) | 26.0 (24.5, 27.5) | 20.5 (18.8, 22.1) |  |
| Q2 | 28.0 (26.2, 29.9) | 27.8 (26.4, 29.2) | 28.7 (26.7, 30.7) |  |
| Q3 | 19.2 (17.8, 20.6) | 19.5 (18.2, 20.7) | 18.4 (16.8, 20.0) |  |
| Q4 | 28.2 (26.8, 29.6) | 26.7 (25.5, 28.0) | 32.4 (30.5, 34.4) |  |
| Energy intake (kcal/day) | 1942.8 ± 12.9 | 2002.1 ± 15.3 | 1772.9 ± 18.5 | < 0.001 |
| Healthy Eating Index | 51.9 ± 0.2 | 51.6 ± 0.3 | 52.6 ± 0.3 | 0.010 |
| Q1 | 22.8 (21.1, 24.4) | 23.5 (22.1, 25.0) | 20.6 (18.7, 22.5) |  |
| Q2 | 24.0 (22.4, 25.6) | 24.3 (22.9, 25.8) | 22.9 (21.3, 24.6) |  |
| Q3 | 25.3 (23.7, 27.0) | 24.3 (23.1, 25.5) | 28.4 (26.4, 30.4) |  |
| Q4 | 27. 9(26.2, 29.7) | 27.8 (26.2, 29.4) | 28.1 (25.9, 30.3) |  |

Values are weighted means ± standard error or weighted % (95% Confidence Interval); p-values are weighted.

^a^ Other races contain Non-Hispanic Asian participants and other non-Hispanic race (including non-Hispanic multiracial).

BMI, Body Mass Index; ASCVD, Arteriosclerotic Cardiovascular Disease; WBC, White Blood Cell Count; NEUT, Neutrophil Cell Count; LYM, Lymphocyte Cell Count; PLT, Platelet Count; HGB, Hemoglobin; RDW, Red blood cell Distribution Width.
